# Selective Alterations of Thiol Redox Homeostasis and Antioxidant Enzyme Activity in Advanced Atherosclerosis

**DOI:** 10.3390/ijms27125496

**Published:** 2026-06-18

**Authors:** Radmil Marić, Branislava Ćurčić, Teodora Vidonja Uzelac, Tanja Grahovac, Zorana Oreščanin Dušić, Srđan Radanović, Danijela Batinić-Škipina, Dragana Drakul

**Affiliations:** 1 Department for Vascular Surgery, University Hospital Foča, 73300 Foča, Bosnia and Herzegovina; radmil.maric@ues.rs.ba (R.M.); srdjanrs1991@gmail.com (S.R.); 2 Faculty of Medicine Foča, University of East Sarajevo, 73300 Foča, Bosnia and Herzegovina; branislava.curcic@ues.rs.ba; 3 Public Health Institution, Hospital Trebinje “St. Archdeacon Stefan—9 January”, 8900 Trebinje, Bosnia and Herzegovina; 4 Department of Physiology, Institute for Biological Research “Sinisa Stankovic”—National Institute of the Republic of Serbia, University of Belgrade, 11000 Belgrade, Serbia; teodora.vidonja@ibiss.bg.ac.rs (T.V.U.); tanja.grahovac@ibiss.bg.ac.rs (T.G.); zoranaor@ibiss.bg.ac.rs (Z.O.D.); 5 ASA Hospital, 71000 Sarajevo, Bosnia and Herzegovina; danijela.batinic-skipina@asabolnica.ba

**Keywords:** oxidative stress, SH groups, atherosclerotic plaque, antioxidant enzyme activity

## Abstract

Atherosclerosis is a progressive vascular disease characterized by lipid-rich plaque accumulation, oxidative stress, and chronic inflammation, contributing to coronary heart disease, stroke, and peripheral arterial disease. This study investigated the impact of inflammation, vascular calcification, and statin therapy on redox balance in blood and carotid artery plaques, aiming to identify potential biomarkers for disease assessment. Thirty-two patients undergoing carotid endarterectomy provided 34 plaque samples. Enzyme activities in plaque/erythrocytes and –SH group concentration in plasma/plaque were measured. Pathological analysis was performed to determine inflammation/calcification grade, the presence of mast cells and plaque composition. The results showed that mast cells were associated with reduced non-protein –SH groups, indicating selective thiol consumption and serving as a qualitative marker of oxidative burden. Reduced catalase activity in erythrocytes was associated with advanced calcification, pointing to long-standing systemic oxidative stress. Statin therapy enhanced systemic superoxide-dismutase 1 activity, increased –SH groups, and modulated plaque-specific glutathione reductase activity, attenuating sex-related differences in redox regulation. These findings highlight the complex interplay between systemic and local oxidative processes in atherosclerosis through alterations in redox-related biomarkers such as plasma –SH group concentrations and catalase activity.

## 1. Introduction

According to the latest estimates, more than 600 million people worldwide are living with some form of cardiovascular disease (CVD) [1]. According to a 2023 analysis, CVD was responsible for approximately 19.2 million deaths, which is 6.1 million more than in 1990 [2]. The absolute number of deaths from CVD continues to rise, partly due to population growth and aging, as well as increased exposure to risk factors.

Among these conditions, atherosclerosis stands out as one of the most common and clinically significant diseases, characterized by the progressive buildup of lipid-rich plaques within arterial walls, which can lead to coronary heart disease, stroke, and peripheral arterial disease. Many factors contribute to the development of plaque; high blood pressure and high levels of low-density lipoprotein (LDL) are the most prominent and well-known risk factors. Statins are most commonly used in the treatment of atherosclerosis, as they effectively lower LDL cholesterol levels and reduce the risk of cardiovascular events [3]. But the antilipemic effects of statins are not the only way by which they exert their antiatherogenic actions. There are animal and human studies that confirm the effects of statins on the vasculature via the suppression of pro-oxidant enzymes as well as stimulation of the AOS [4,5].

Over the past three decades, oxidative stress and inflammation have been identified as important pathogenic processes in atherosclerotic CVD. It is known that artery remodeling during the atherosclerosis process is associated with LDL oxidation, creating a further increase in inflammatory cells and proteolytic enzymes at the lesion site [6,7]. Also, there is an increase in reactive oxygen species (ROS) and oxidative stress in artery walls [5,8].

Endothelial dysfunction represents an early and pivotal event in the development of atherogenesis. It disrupts the balance between vasoconstriction and vasodilation, increases endothelial permeability, and initiates a local inflammatory response. This leads to the infiltration of circulating inflammatory cells into the vascular wall, accompanied by the induction of cytokines and other inflammatory mediators [9,10]. Concurrent recruitment of monocytes/macrophages, mast cells, and T lymphocytes into the intima further amplifies inflammation and promotes the production of ROS [11].

Vascular calcification is characteristic of advanced atherosclerosis. It leads to narrowing of the lumen and a reduction in the elasticity of the blood vessel [12]. Vascular smooth muscle cells play a key role in the progression of atherosclerotic lesions by enhancing their migration and proliferation, increasing the production of extracellular matrix components, and undergoing osteogenic differentiation that promotes vascular calcification [13]. Numerous conditions associated with a higher incidence of vascular calcification are also characterized by increased oxidative stress, such as hypercholesterolemia, hypertension, diabetes mellitus, and end-stage renal disease requiring dialysis [14].

Processes in carotid plaque development include ROS [15], but the role and involvement of antioxidative defense in carotid plaque are not known. The normal function and structure of arteries, as well as other tissues, are dependent on a vigilant anti-oxidative defense system (AOS) capable of maintaining a balance between constantly forming pro-oxidative factors, ROS, and anti-oxidative processes. Enzymes and molecules of AOS constantly neutralize ROS and other molecules, with oxidative effects generated through various pathways [16]. The main cellular components of the AOS include enzymatic antioxidants such as superoxide dismutase 1 (SOD1), superoxide dismutase 2 (SOD2), catalase (CAT), glutathione peroxidase (GPx), and glutathione reductase (GR), as well as non-enzymatic components, including glutathione (non-protein thiol groups) and protein thiol groups.

Accordingly, this study was designed to investigate the impact of inflammation, vascular calcification, and statin therapy on oxidative balance in both blood and carotid artery plaques of patients with atherosclerosis.

## 2. Results

### 2.1. Study Sample

The demographic and clinical characteristics of the participants and the overall study sample are shown in Table 1. Homogeneity of variances for continuous variables was confirmed using Levene’s test. During the 5-year follow-up period, 32 patients who underwent CEA were monitored, including 21 men and 11 women. Five patients died during follow-up, corresponding to an overall mortality rate of 15.6%. Mortality was numerically higher among men (19.0%) compared with women (9.1%); however, no statistically significant sex-related difference in mortality was observed. All recorded deaths were attributable to cardiovascular events.

Four patients (12.5%) were lost to follow-up, including two men and two women. Among the remaining patients who completed follow-up evaluation, clinical status remained stable, and no cases of carotid restenosis were identified during the observation period.

### 2.2. Pathological Characteristics of Carotid Plaques

**Figure 1:**
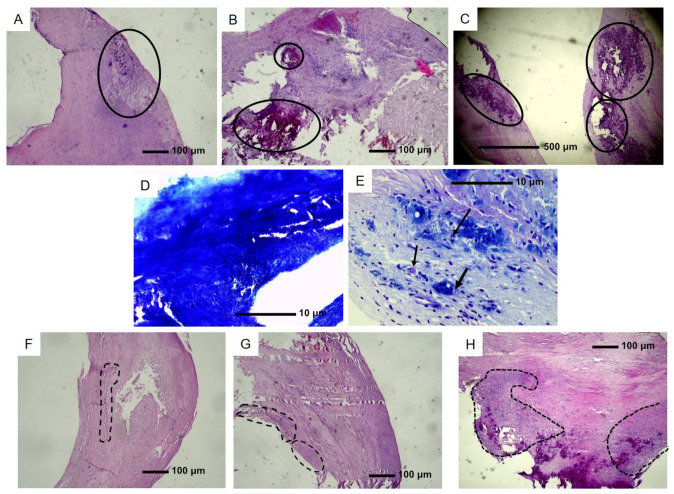
Carotid plaque characteristics. (**A**–**C**) Calcifications in carotid plaque, marked with a closed black solid line, grade I–III, respectively (hematoxylin–eosin staining, magnification 100×); (**D**) plaque without mastocytes (Giemsa staining, magnification 200×); (**E**) mastocytes in carotid plaque, marked with black arrows (Giemsa staining, magnification 400×); (**F**–**H**) inflammation in carotid plaques, marked with a black dotted line, grade I–III, respectively (hematoxylin–eosin staining, magnification 100×).

Plaque characteristics such as calcification grade, the presence of mast cells and inflammation level are presented in Figure 1.

### 2.3. Antioxidant Enzyme Activities and Concentration of SH Groups in Plaque and Blood Samples

**Figure 2:**
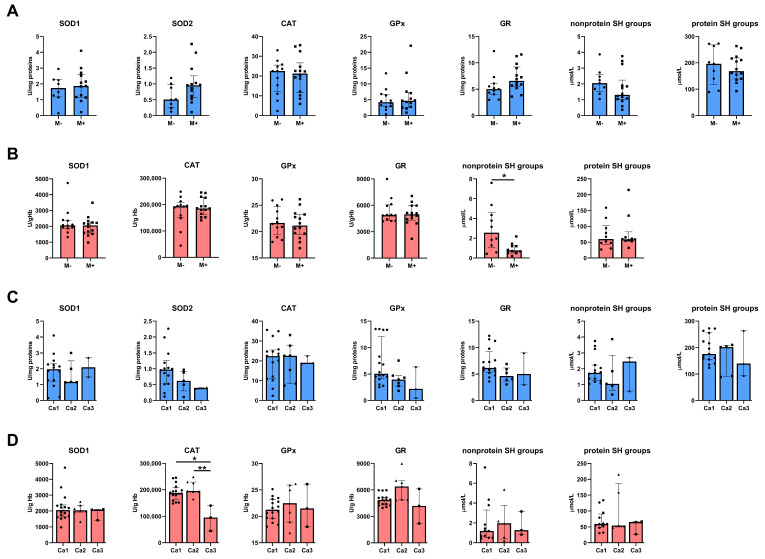
The activity of antioxidant enzymes and concentrations of protein and non-protein SH groups in the plaque (**A**) and blood (**B**) based on the absence (M−) or presence (M+) of mast cells within the plaque. The activity of antioxidant enzymes and concentrations of protein and non-protein SH groups in the plaque (**C**) and blood (**D**) based on calcification grade: grade I (microcalcifications)—less than half of the plaque volume (Ca1); grade II—calcifications occupied approximately half of the plaque volume (Ca2); and grade III—more than half of the plaque volume (Ca3). The results are expressed as the median and interquartile range and individual data points have been added to the figures (squares, triangles, and dots). Statistical significance was calculated by the Mann–Whitney U test for pairwise comparisons (presence of mast cell). The Kruskal–Wallis ANOVA was use to compare central tendencies among three independent groups (level of calcification in plaque), and a post hoc Dunn’s multiple comparisons test was performed to determine which groups differed from each other. The overall level of statistical significance for all analyses was set at *p* < 0.05. SOD1—superoxide dismutase 1; SOD2—superoxide dismutase 2; CAT—catalase; GPx—glutathione peroxidase; GR—glutathione reductase; *—*p* < 0.05, **—*p* < 0.01.

The presence of mast cells within the plaque in our study was associated with reduced plasma levels of non-protein –SH groups (Mann–Whitney U test, *p* = 0.0232, effect size r = 0.51), as shown in Figure 2B. There were no statistically significant changes in the level of protein –SH groups or the activity of antioxidant enzymes in erythrocytes (Figure 2B), as well as in measured parameters in plaque, as shown in Figure 2A.

Our results showed a decrease in catalase activity in the erythrocytes of patients with advanced plaque calcification (Kruskal–Wallis test, *p* = 0.0115, effect size ε^2^ = 0.3, post hoc Dunn’s multiple comparisons test Ca1 vs. Ca3 *p* = 0.0423; Ca2 vs. Ca3, *p* = 0.0087), as shown in Figure 2D. There were no statistically significant differences in catalase activity between the Ca1 and Ca2 groups. Additionally, no statistically significant changes were observed in the levels of –SH groups or in enzyme activities in plaque samples, as shown in Figure 2C.

**Figure 3:**
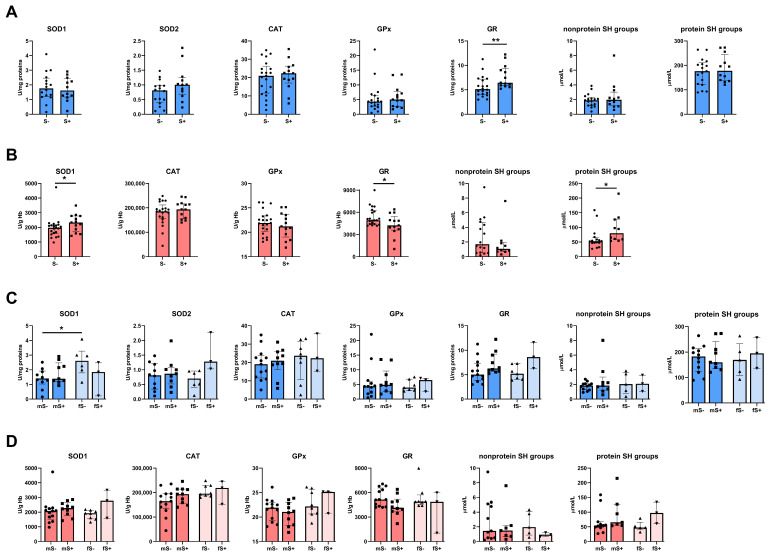
The activity of antioxidant enzymes and concentrations of protein and non-protein SH groups in the plaque (**A**) and blood (**B**) based on statin use regardless of sex: non-statin use (S−) and statin use (S+). The activity of antioxidant enzymes and concentrations of protein and non-protein SH groups in the plaque (**C**) and blood (**D**) based on statin use and sex, male non-statin use (mS−), male statin use (mS+), female non-statin use (fS−), and female statin use (fS+). The results are expressed as yje median and interquartile range and individual data points have been added to the figures (squares, triangles, and dots). Statistical significance was calculated by the Mann–Whitney U test for pairwise comparisons (statin use regardless of sex). The Kruskal–Wallis ANOVA was used to compare central tendencies among four independent groups (statin use based on sex). In addition, the Mann–Whitney U test was used to compare men and women who did/did not use statins. The overall level of statistical significance for all analyses was set at *p* < 0.05. SOD1—superoxide dismutase 1; SOD2—superoxide dismutase 2; CAT—catalase; GPx—glutathione peroxidase; GR—glutathione reductase; *—*p* < 0.05, **—*p* < 0.01.

When patients were grouped solely according to statin use, regardless of sex, GR activity in plaque samples was significantly increased in statin users (Mann–Whitney U test, *p* = 0.0071, effect size r = 0.46, Figure 3A). In the same group, erythrocyte samples showed increased SOD1 activity (*p* = 0.0461, effect size r = 0.34) and decreased GR activity (*p* = 0.0292, effect size r = 0.37), accompanied by elevated plasma protein sulfhydryl (–SH) levels (Mann–Whitney U test, *p* = 0.0144, effect size r = 0.47, Figure 3B).

The Kruskal–Wallis ANOVA was used to compare central tendencies among four independent groups (when patients were categorized by sex), without statistical significance. However, higher SOD1 activity (Mann–Whitney U test, *p* = 0.042, effect size r = 0.51) was observed in plaque samples from women compared with men not receiving statin therapy (Figure 3C). There were no changes in the activity of other antioxidant enzymes and SH groups in plaques, as shown in Figure 3C. Also, there were no changes in erythrocyte samples, as shown in Figure 3D.

Plaque classification into fibrous and fibrolipidic types, level of inflammation (I–III), and smoking did not show significant differences in the activity of antioxidant enzymes and the concentration of SH groups in the plaque and erythrocyte samples of atherosclerotic patients (Table S1, Supplementary Materials).

## 3. Discussion

A large number of studies indicate that parameters of tissue redox status may serve as markers of CVD. Classical antioxidant enzymes (SOD, GPx, and CAT) play an important role in protecting blood vessels, but their activity in coronary artery disease varies and depends on the stage of the disease [17]. Total antioxidant status is reduced in patients [18], while elevated MDA levels are associated with risk factors and the progression of atherosclerosis, making it an independent predictor of cardiovascular events [19]. Additionally, oxidized LDL formed through the action of ROS contributes to the development of atherosclerosis and has significant predictive value for cardiovascular outcomes [20].

The presence of mast cells within the plaque was associated with reduced plasma levels of non-protein –SH groups in our study, suggesting selective consumption of thiol antioxidants within a specific oxidative microenvironment. Mast cells are known to release proteases and mediators that can enhance local production of ROS [21], thereby promoting the initial depletion of low-molecular-weight thiols as a first line of redox defense [22,23]. Nevertheless, this association was observed only in plasma and was not confirmed by plaque biochemical parameters, suggesting that –SH groups may be an indirect rather than a direct marker of local oxidative processes. These findings suggest that mast cells may serve as a potential qualitative indicator of oxidative burden within the plaque. This highlights the potential value of thiol status as a more sensitive indicator of oxidative alterations compared to antioxidant enzyme activity alone. Although they are measured in plasma, –SH groups are an indirect indicator of redox status in tissues, including the vascular wall and the atherosclerotic plaque. This is because there is a constant exchange of thiol compounds between plasma and tissues [24,25,26]. In atherosclerosis, glutathione (GSH) is oxidized to GSSG, the availability of free –SH groups is reduced, and tissue shifts toward a pro-oxidative and pro-inflammatory state [27]. Plasma –SH levels thus become a marker of systemic redox imbalance, as well as an indirect indicator of depletion of the glutathione system in the vascular wall.

Histological grade of inflammation was not accompanied by changes in antioxidant enzyme activity in the plaque or erythrocytes of atherosclerotic patients, indicating that the quantity of the inflammatory infiltrate does not necessarily reflect the functional intensity of oxidative stress within the lesion. However, the assessment of inflammation was semiquantitative and based solely on cell number, without distinguishing between different inflammatory cell phenotypes or their metabolic activity, which strongly limit the sensitivity of this approach. It is possible that in chronic atherosclerosis, long-term adaptation of the redox system occurs, leading to the stabilization of enzymatic activity at a new homeostatic level, independent of variations in the degree of inflammation. Alternatively, different inflammatory cell populations may exert divergent effects on redox balance, which cannot be captured by grading inflammation alone.

The selective decrease in CAT activity observed in erythrocytes of patients with advanced plaque calcification in our study likely reflects the unique role of CAT in detoxifying hydrogen peroxide under conditions of chronic oxidative stress. In advanced atherosclerosis, hydrogen peroxide represents a key redox mediator promoting osteogenic differentiation and vascular calcification [28]. Unlike other antioxidant enzymes, CAT acts as a high-capacity enzyme for H_2_O_2_ removal and is particularly susceptible to oxidative inactivation [29]. While SOD and GPx activities may remain preserved due to compensatory mechanisms or inducible regulation [30], prolonged exposure to elevated H_2_O_2_ levels may lead to selective exhaustion or inactivation of CAT [31]. Therefore, the observed reduction in CAT activity may represent a marker of long-standing redox imbalance associated with advanced vascular calcification rather than a generalized impairment of the antioxidant defense system. The absence of similar changes in plaque tissue may reflect either local compensatory mechanisms or methodological limitations related to tissue homogenization, which can mask focal oxidative alterations. The finding that the decrease in CAT activity is observed in erythrocytes, but not in the plaque tissue of patients, indicates that advanced calcification is not merely a local phenomenon but a systemic one, and that patients with grade III calcification exhibit a globally impaired redox status. This suggests that the degree of plaque calcification carries a systemic antioxidant signature rather than having solely morphological significance.

Statins exert pleiotropic effects, which means that, in addition to lowering cholesterol, they have additional cholesterol-independent actions, such as anti-inflammatory, antioxidant, endothelium-protective, and antithrombotic effects, which contribute to plaque stabilization and slow the progression of atherosclerosis [32]. Their redox effects are often local, subtle and they depend on dose, duration of therapy, and individual response [33].

When patients were analyzed irrespective of sex, statin therapy was associated with increased SOD1 activity in erythrocytes, accompanied by decreased GR activity and elevated levels of protein sulfhydryl (–SH) groups in blood. The increase in SOD1 activity suggests enhanced dismutation of superoxide radicals, indicating activation of primary antioxidant defense mechanisms. In contrast, the observed reduction in GR activity may reflect a decreased demand for glutathione regeneration due to lower oxidative burden. However, alternative explanations, such as differential regulation or inhibition of GR activity, cannot be excluded. This indicates a shift in redox balance toward a less oxidative state. Furthermore, the increase in protein –SH groups supports this interpretation, as thiol groups are highly susceptible to oxidative modification. Their preservation suggests reduced protein oxidation and improved redox homeostasis. Taken together, these findings indicate that statin therapy can be associated with stabilization of systemic redox balance by enhancing primary antioxidant capacity while simultaneously reducing the need for compensatory glutathione-dependent mechanisms. This pattern is consistent with a lower oxidative stress state rather than impaired antioxidant defense. In the plaque, however, the situation is different: the local tissue is exposed to chronic inflammation and increased ROS production, necessitating enhanced antioxidant defense. Therefore, in patients receiving statins, an increase in GR activity is observed in the plaque. This represents an adaptive response, as GR regenerates GSH from GSSG, thereby strengthening local antioxidant protection and contributing to plaque stabilization. Thus, while statins are associated with a systemic reduction in oxidative stress (in the blood), they are related with local activation of protective mechanisms within the plaque.

Additionally, when patients were categorized by sex, we compared men and women receiving statin therapy with their respective counterparts who were not treated with statins. Higher SOD1 activity was observed in plaque samples from women compared with men not receiving statin therapy. Estrogen has well-documented antioxidant and SOD-inducing effects, and vascular tissue in women exhibits stronger basal redox adaptation [34]. Therefore, the elevated SOD1 activity in women not treated with statins may reflect an endogenous, hormone-mediated compensatory mechanism. The difference in SOD1 activity between men and women was not observed in the group using statins, likely because statins partially assume the antioxidant role and may neutralize sex-related differences in redox adaptation. Statins have dose-dependent pleiotropic effects, and without detailed stratification according to statin type, dose, and treatment duration, statin-related findings should be taken with caution and warrant further investigation.

Our results did not show a significant effect of smoking on the activity of antioxidant enzymes and concentrations of SH groups in the plaque and erythrocytes of atherosclerotic patients. This can be explained by the fact that smoking has a strong effect in the early stages of the disease. Its impact is cumulative and time-dependent. In patients with advanced atherosclerosis, the effect of smoking is often masked by the background of chronic oxidative stress [35].

The classification of plaques into fibrous and fibrolipidic types also did not show differences in the enzyme activity and concentrations of SH groups in the plaque and erythrocyte samples. This is because this morphological classification does not necessarily reflect metabolic or oxidative activity and does not distinguish plaques based on the intensity of inflammation or ROS production. Future studies using molecular or functional markers of plaque activity may provide a more precise characterization of redox-related changes than morphology alone.

## 4. Study Limitations

A major limitation of the present study is the small sample size and unequal sex distribution, partly due to the single-center design. This limited the availability of a larger patient cohort and consequently resulted in the lack of detailed stratification by statin type, dose, and treatment duration—an aspect that must be considered when interpreting these findings. Also, not all patients attended regular follow-up appointments. This may reduce statistical power and limit the detection of subtle effects. The fact that two patients contributed bilateral plaque samples may partially affect the assumption of independence of observations and therefore represents a methodological limitation of the study. Therefore, our findings require validation in larger, multicenter studies.

## 5. Materials and Method

The study protocol was approved by the local ethics committee of UHF (01-942/2-2). All patients participated in this study on a voluntary basis and by signing the informed consent form. Data on demographic characteristics were collected from participants’ medical histories and by means of a questionnaire designed for study purposes.

In this study, 32 patients were involved that had undergone carotid endarterectomy (CEA) at University Hospital Foča between June 2019 and March 2020. Two of those patients underwent CEA two times during that period, for left and right carotid arteries, separately. Thus, 34 human samples (man—23; women—11) of carotid artery plaques were collected. The patients were followed up for five years after surgery to assess postoperative outcomes and long-term clinical status. Each sample was assigned a number in order to ensure the anonymity of the study participants.

After dissection and flushing in 0.9% NaCl to remove blood residue, each plaque was split into two approximately equal parts immediately. One half was embedded in 4% formaldehyde, put in paraffin blocks, and sectioned with a microtome at 5 µm thickness. The other part was instantly frozen in liquid nitrogen and kept at −80 °C until biochemical analysis. The activity of antioxidant enzymes in plaque and of erythrocytes, as well as the concentration of sulfhydryl (SH) groups in plasma and plaque samples, was measured.

The effects of inflammation, the presence of mast cells, calcification, smoking, and statin therapy in all patients, regardless of sex, were evaluated. The effect of statin therapy was also analyzed according to patients’ sex.

Carotid artery ultrasound was performed on all patients preoperatively and all patients had >70% stenosis. The average maximum peak in-flow velocity of the internal carotid artery was >175 cm/s. A detailed medical history was taken from each patient with special attention on atherosclerosis-connected factors.

### 5.1. The Pathological Analysis of Carotid Plaques

Pathological analysis was performed to determine if plaque samples have primarily fibrous or fibrolipid composition. Histopathological evaluation was performed by an experienced pathologist blinded to the clinical and laboratory data of the patients. Calcification grade was determined in a semiquantitative manner (hematoxylin–eosin staining, magnification 100×) by rating calcium content from I to III. It was assessed on the entire histological plaque specimen at medium magnification and graded according to the proportion of calcium deposits relative to the total plaque volume as follows: grade I (microcalcifications), in which calcifications occupied less than half of the plaque volume; grade II, in which calcifications occupied approximately half of the plaque volume; and grade III, in which calcifications occupied more than half of the plaque volume. Mast cells were evaluated on the entire plaque specimen at high magnification (400×). Due to their low abundance, mast cells were classified qualitatively as present or absent. Inflammatory cells were counted in two representative high-density fields per 1 mm^2^ at medium magnification in the plaque regions showing the greatest inflammatory cell infiltration. Inflammation levels in plaque samples (hematoxylin–eosin staining, magnification 100×) ranged as follows: grade I (non to 10 inflammatory cells/mm^2^ on 400× microscopic field); grade II (10–30 inflammatory cells/mm^2^); grade III (>30 inflammatory cells/mm^2^).

### 5.2. Determination of Antioxidant Enzyme Activities in Carotid Plaques

Thawed carotid plaques samples were weighed, homogenized, and sonicated in 0.25 M sucrose, 1 mM ethylenediaminetetraacetic acid, and 0.05 M Tris-HCl buffer (pH 7.4) before centrifugation for 90 min at 105,000× *g*. The supernatant was used to determine antioxidant enzyme activities and the concentration of non-protein and protein –SH groups, as described previously [36,37].

Briefly, total superoxide dismutase (SOD) activity was determined by using the adrenaline method [38]. One SOD unit was defined as the amount of the enzyme necessary to decrease the rate of adrenalin auto-oxidation by 50% at pH 10.2. For the determination of SOD2 activity, the assay was performed after preincubation with 8 mM KCN. The SOD1 activity was calculated as the difference between the total SOD and SOD2 activities. CAT activity was determined by the monitoring of hydrogen peroxide consumption [39]. The GPx activity is based on the reduction in GSH by using t-butyl-hydroperoxide as a substrate [40]. GR activity was determined by NADPH oxidation concomitant with GSSG reduction [41]. All enzyme activities were expressed in U/mg protein. Protein concentration was measured by using the method of Lowry [42].

Before surgery, whole blood samples of each patient were taken in heparinized vacutainers. Erythrocytes and plasma were immediately separated by centrifugation (10 min, 5000 rpm, 20 °C). Aliquots of erythrocytes were washed three times with 0.9% NaCl and lysed in ice-cold distilled water. Antioxidant defense enzyme activities were measured in the lysate. For the determination of SOD activity, hemoglobin was removed by using the method of Tsuchihashi [43] and values were estimated by using the method of Drabkin and Austin [44]. Enzyme activity was measured in the same way as in plaques only expressed in U/g of Hb [45].

### 5.3. Determination of the Concentrations of –SH Groups

The concentrations of the total—SH groups were measured according to Ellman’s protocol customized for microtiter plates [46]. Samples were mixed with Ellman’s reagent (5,5-dithiobis-(2-nitrobenzoic acid)) and after 10 min the absorbance was measured at 412 nm. For the determination of non-protein –SH groups, proteins were precipitated by the addition of sulfosalicylic acid. Protein –SH groups were calculated as the difference between total and non-protein –SH groups.

### 5.4. Statistical Analyses

Differences between independent experimental groups were analyzed using non-parametric statistical methods due to the unequal number of samples in each group. First, a Kruskal–Wallis ANOVA was applied to compare central tendencies among three or more independent groups. If the Kruskal–Wallis test indicated a statistically significant difference (*p* < 0.05), a post hoc Dunn’s multiple comparisons test was performed to determine which groups differed from each other. The Mann–Whitney U test was used for pairwise comparisons. The overall level of statistical significance for all analyses was set at *p* < 0.05. Demographic data were assessed using Student’s *t*-test for continuous variables and Fisher’s exact test for categorical variables. Homogeneity of variances for continuous variables was tested using Levene’s test. Statistical analyses were performed using Graph Pad Prism 8.

## 6. Conclusions

This study provides insight into the complex relationship between oxidative stress, plaque characteristics, and clinical factors in advanced atherosclerosis. The findings suggest that plasma –SH levels may be a marker of systemic redox imbalance while mast cells may serve as a potential qualitative indicator of oxidative burden within the plaque. Reduced CAT activity in erythrocytes associated with advanced calcification points to long-standing systemic oxidative stress, suggesting that atherosclerosis has a significant systemic component and indicating that CAT activity may serve as marker of oxidative stress and antioxidant response. The results show that statin therapy is associated with enhanced systemic and plaque-specific antioxidant defense, while also influencing the attenuation of inherent sex-related differences in redox regulation, indicating that its antioxidative effects may partially override endogenous, hormone-mediated compensatory mechanisms. However, future studies with larger cohorts should investigate the effects of statins while carefully considering sex-specific differences. Taken together, these findings highlight the interplay between systemic and local redox processes in atherosclerosis.

## Figures and Tables

**Table 1:** The demographic and clinical characteristics of the participants regarding gender difference.

Variable	Men (n = 21)	Women (n = 11)	*p* Value
Age (mean ± SD)	68.2 ± 6.4	66.5 ± 8.2	0.514
5 years survival	15 yes—4 no	8 yes—1 no	0.865
Diabetes mellitus, n (%)	3 (14.3%)	1 (9.1%)	0.573
Hypertension, n (%)	18 (85.7%)	10 (90.9%)	0.573
Overweight, n (%)	2 (9.5%)	3 (27.3%)	0.209
Smoking, n (%)	13 (61.9%)	4 (36.4%)	0.158
Alcohol consumption, n (%)	6 (28.6%)	0 (0.0%)	0.071

## Data Availability

Data are available at https://radar.ibiss.bg.ac.rs/handle/123456789/8098 (accessed on 27 May 2026).

## Supplementary Materials

The following supporting information can be downloaded at: https://www.mdpi.com/article/10.3390/ijms27125496/s1.
