# Supplementary material for: Selective Alterations of Thiol Redox Homeostasis and Antioxidant Enzyme Activity in Advanced Atherosclerosis
Source: Int J Mol Sci. 2026 Jun 18;27(12):5496. doi: 10.3390/ijms27125496 (PMC13300297; doi:10.3390/ijms27125496)
Supplement: Supplementary file 1 [file ijms-27-05496-s001.zip › ijms-4319848-supplementary.pdf]

Table S1

		SOD1	SOD2	CAT	GPx	GR	nonprotein SH groups	protein SH groups
plaque	**I1**	1.38 (0.90-2.07)	0.70 (0.47-1.05)	20.80 (10.11-26.29)	4.24 (2.50-6.48)	5.02 (3.54-6.43)	2.24 (1.38-2.72)	202.16 (94.21-272.07)
	**I2**	1.86 (1.16-2.17)	0.88 (0.45-1.61)	22.60 (19.30-32.54)	4.78 (4.03-8.32)	6.26 (5.75-9.82)	1.38 (1.04-1.85)	205.24 (155.20-223.62)
	**I3**	2.70 (1.72-3.52)	0.87 (0.27-0.98)	18.71 (8.06-22.47)	2.90 (2.35-4.78)	5.70 (4.79-8.32)	1.90 (0.84-2.75)	159.91 (131.97-172.71)
blood	**I1**	2060.03 (1909.47-2478.95)		193697.05 (134993.71-216391.87)	21.64 (20.32-25.24)	4914.04 (4231.89-6291.63)	1.96 (0.92-4.57)	52.28 (39.00-110.57)
	**I2**	2141.49 (1590.68-2397.77)		180045.15 (158529.35-229573.50)	21.58 (18.77-23-45)	5095.48 (4819.10-5966.99)	0.93 (0.43-1.63)	58.44 (54.15-154.21)
	**I3**	1743.34 (1519.90-2258.27)		185650.79 (162606.33-195716.96)	20.60 (18.88-22.62)	4405.36 (3070.37-4772.48)	0.82 (0.61-2.77)	59.31 (45.45-73.35)
plaque	**smo-**	1.57 (1.15-2.34)	0.85 (0.48-0.97)	20.98 (8.03-26.21)	4.53 (3.46-6.71)	5.73 (4.82-7.30)	1.88 (1.16-2.86)	200.03 (161.02-226.46)
	**smo+**	1.92 (1.27-2.54)	1.01 (0.48-1.21)	22.30 (17.05-26.25)	4.64 (2.29-7.87)	6.08 (4.54-8.80)	1.92 (1.29-2.32)	140.05 (120.47-203.09)
blood	**smo-**	2041.75 (1707.43-2214.04)		188968.09 (172311.65-229530.02)	22.47 (20.17-24.23)	4909.16 (4360.65-5548.97)	1.20 (0.54-3.47)	56.08 (47.668-100.57)
	**smo+**	2133.94 (1564.78-2587.42)		192825.95 (149973.002-213072.92)	21.46 (19.27-23.06)	4564.74 (3883.38-6042.24)	1.44 (0.75-4.73)	60.80 (51.11-95.88)
plaque	**F**	1.49 (1.15-2.24)	0.52 (0.37-1.01)	22.51 (10.98-25.82)	4.52 (2.73-6.83)	5.11 (3.80-6.38)	2.01 (1.45-2.66)	199.20 (105.68-270.09)
	**FL**	1.97 (1.16-2.77)	0.91 (0.50-1.14)	22.30 (12.09-24.74)	4.51 (2.72-7.22)	6.26 (5.22-9.01)	1.38 (1.04-2.05)	169.40 (139.99-2126.70)
blood	**F**	2078.30 (1923.34-2397.77)		192825.95 (148230.79-212026.98)	21.47 (18.93-25.01)	4909.16 (4234.29-6117.49)	1.96 (0.92-4.57)	52.28 (39.00-110.57)
	**FL**	1975.25 (1590.68-2241.44)		185650.79 (164687.59-225956.77)	21.58 (19.62-23.26)	4854.83 (4405.36-5961.19)	0.82 (0.51-1.44)	59.31 (54.54-73.30)

Table S1. Activity of antioxidant enzymes and concentrations of protein and non-protein SH groups in the plaque and blood according to clasification into fibrous and fibrolipidic types of plaque, smoking status, as well as level of inflamation in plaque. SOD1 – superoxide dismutase 1, SOD2 – superoxide dismutase 2, CAT – catalase, GPx – glutathione peroxidase, GR – glutathione reductase; I1 – inflammation level: grade I, I2 – inflammation level: grade II, I3 – inflammation level: grade III; smo- – non-smokers, smo+ – smokers; F – fibrous composition, FL – fibrolipid composition. The results are expressed as the median (interquartile range). All enzyme activities are expressed in U/mg of protein for plaque or U/gHb for blood. Concentration of SH goups are expresed in μmol/L. Statistical significance was calculated by the Mann–Whitney U test for pairwise comparisons (types of plaque) and the Kruskal–Wallis ANOVA to compare central tendencies among three independent groups (level of inflamation in plaque). The overall level of statistical significance for all analyses was set at $p < 0.05$.
